# Contextual information in medicolegal death investigation decision-making: Manner of death determination for cases of a single gunshot wound

**DOI:** 10.1016/j.fsisyn.2022.100285

**Published:** 2022-09-03

**Authors:** Itiel E. Dror, Dwayne A. Wolf, Garrett Phillips, Si Gao, Yijiong Yang, Stacy A. Drake

**Affiliations:** a University College London, United Kingdom; b Harris County Institute of Forensic Science, USA; c Bexar County Medical Examiner's Office, USA; d The University of Texas Health Science Center at Houston, USA; e Texas A&M University, USA

**Keywords:** Forensic pathology, Manner of death, Cognitive bias, Contextual information, Forensic science

## Abstract

To explore the role of contextual information in determining manner of death, four cases involving single gunshot wounds were presented to participants (n = 252) involved in medicolegal death investigation. The participants received identical autopsy information but different contextual information. The data demonstrated that participants tended to rely on contextual information more than autopsy information: In the suicide context, participants across the four cases reached 153 final decisions of suicide (and 25 of homicide), whereas in the homicide context, participants reached only 10 final decisions of suicide (and 181 of homicide) --all while examining identical autopsy information. The impact of the contextual information was so powerful that many participants changed initial autopsy-based conclusions to align with the contextual information. Given the significant role and impact that contextual information has on expert decision making, one must consider what, how, and when contextual information should be used.

## 1 Introduction

Medicolegal death investigations impact public health policies by providing statistical data about manners of death, and can play a critically important role within the criminal justice system [1]. Although death investigation systems vary, a unifying feature of many is the classification of cause and manner of death, which are reported on death certificates. Relevant contextual information is often necessary to determine manner of death. However, the use of contextual information can be perilous, as it varies in its level of objectivity, biasability and relevance. Inappropriately prioritizing or trivializing a given piece of contextual information can lead to errors in manner of death determinations. The cognitive processes, some of them without intention or awareness, of incorporating or ignoring (or emphasizing or de-emphasizing) portions of information are therefore of vital importance.

The use of contextual information presents a potential source of cognitive bias. We emphasize *cognitive* bias to distinguish it from the lay everyday usage of the term bias (an intentional discriminatory bias, such as sexism, racism, or antisemitism). Cognitive bias, well established to occur in many areas of expert decision making, refers to unintentional implicit bias that impacts even hard working, competent and dedicated professionals [2,3].

A substantial amount of research has demonstrated specifically that medical doctors are prone to such cognitive bias (e.g., [[4], [5], [6], [7]]. Research has also demonstrated that forensic science domains are not immune to the effects of cognitive bias, including fingerprinting, DNA and toxicology [8,9].

Moreover, a recent paper investigating cognitive bias specifically in medicolegal death investigation suggests that manner of death expert decisions are also susceptible to cognitive bias, as are other forensic domains and medical decision making [10]. In their 2021 study, 133 participants receiving identical medical information regarding an injured child nevertheless reached different manner of death determinations (accident vs. homicide) due to the impact of contextual information characterized as non-medical and irrelevant.

Using irrelevant or erroneous contextual information can cause errors in manner of death determinations. Such errors may waste police resources, and even contribute to a wrongful conviction. Conversely, they may lead to the erroneous conclusion that no investigation is warranted, which may allow a murder to go undetected.

The impact of contextual information depends not only on its content, but also on other cognitive factors, such as who provides it, how, and when it is given (e.g., the information sequencing can impact when and which expectations and hypotheses are generated).

Given the role and impact that contextual information has on expert decision making in general, and on forensic pathology specifically, one must consider what, how, and when contextual information should be used [11]. Methods intended to optimize the use of contextual information should be informed by an understanding of how contextual information influences decisions. Accordingly, the present study sought to address two questions: First, how are contextual information and autopsy findings used in manner of death determination? Second, what is the impact of the order in which information is presented (context first, autopsy second, or vice versa) on manner of death decisions?

## 2 Methods

### 2.1 Design and materials

The experiment utilized a survey design and included four cases investigated by the Harris County Institute of Forensic Sciences (HCIFS). All the cases had a single gunshot wound (with locations including the head, neck, or torso) as the cause of death. The autopsy information for each of the four cases consisted of photographs which were selected by practicing forensic pathologists to adequately demonstrate the characteristics of the wounds (5–9 autopsy photographs were presented per case).

All participants, regardless of contextual information, received identical autopsy photographs for each case. Contextual information (a narrative history and scene photographs) for each of the four cases was developed to suggest either a suicidal or homicidal manner of death (see Table 1 for sample suicide and homicide narratives). The scene photographs were selected, and in some instances modified, to support a determination of suicide or homicide (e.g., a gun under the decedent's body digitally removed or cropped out). The scene photographs were then paired and matched with the corresponding narratives to support the same manner of death determination –for the contextual information, participants received the narrative history first and then the scene photographs. Hence, the experiment was a 2 (suicide/homicide context) x 2 (order of presentation) x 4 (number of cases) factorial design.

**Table 1:** Sample narratives.

	Suicidal context	Homicidal context
Case A1	29 year old Hispanic female who was a security guard at a warehouse. The decedent reported for work at her scheduled time. She was approached by a coworker and was reported to be very quiet and to herself. The decedent reportedly went to the bathroom at around 2:00AM. She exited the bathroom −2:20AM with a handgun and shot one of her coworkers. The decedent then walked outside of the warehouse where she met several law enforcement officers responding to the warehouse. A brief exchange of gunfire occurred, after which time the decedent sheltered behind a car on the parking lot and officers lost sight of her, although they then heard a single gunshot. The decedent was subsequently found face down outside of the warehouse in the parking lot with a single gunshot wound of the right posterior head. She was transported to a hospital and pronounced dead shortly after arrival. The decedent's spouse reported that the decedent had been involved in criminal activity −12 years ago with a group of coworkers and was never caught. The decedent reportedly became paranoid that the individuals she committed the crimes with were going to ‘rat on her’. The employee that was shot by the decedent was reportedly one of the accomplices in the crimes several years ago.	29 year old Hispanic female who was a security guard at a warehouse. The decedent reported for work at her scheduled time. She was approached by a coworker and was reported to be very quiet and to herself. The decedent reportedly went to the bathroom at around 2:00AM. She exited the bathroom −2:20AM with a handgun and shot one of her coworkers. The decedent then walked outside of the warehouse where she met several law enforcement officers responding to the warehouse. The decedent was verbally commanded to drop her weapon, but she refused to comply and reportedly began to walk away from officers. As they continued to command her to drop the weapon, the decedent partially turned and was witnessed to begin to raise her hand with the gun, at which time officers opened fire on her. Officers then approached the decedent and found her to have sustained a single gunshot wound of the right posterior head. She was transported to a hospital and pronounced dead shortly after arrival. The decedent's spouse reported that the decedent had been involved in criminal activity −12 years ago with a group of coworkers and was never caught. The decedent reportedly became paranoid that the individuals she committed the crimes with were going to ‘rat on her’. The employee that was shot by the decedent was reportedly one of the accomplices in the crimes several years ago.

## 3 Procedure

Each participant in this online study received all four cases. The contextual information for each of the four cases (either suggesting a suicide or a homicide) was presented to the participants either before or after of the autopsy information in each case (see Table 2). After each phase (presenting context or autopsy), the participants were asked to provide their opinion about the manner of death. They could decide the manner of death was suicide, homicide, accident, or natural, and if they could not decide, they could state that it was undetermined. Participants were not able to change their decision. After phase 1, the next set of information was presented in phase 2 (if in phase 1 they got the autopsy information, then in phase 2 they got the contextual information, and vice versa). After phase 2 information, participants again gave their conclusion about the manner of death. This process was repeated sequentially for each of the four cases.

**Table 2:** Experimental Design. All participants received identical autopsy information for each of the four cases that were presented. The participant groups were created by varying when the contextual information was received (either before or after the autopsy information), and whether they received contextual information suggesting homicide (H) or suicide (S). Thus, the four groups, labeled by their experimental conditions, were: Group A-H (**A**utopsy information first, then **H**omicide context), Group A-S (**A**utopsy information first, then **S**uicide context), Group H-A (**H**omicide context first, then **A**utopsy information), and Group S-A (**S**uicide context first, then **A**utopsy information). After each phase, participants provided a determination of the manner of death: suicide, homicide, accident, natural, or undetermined.

Groups	Phase 1	Participant Decision	Phase 2	Participant Decision
A-H	Autopsy First	?	Context Second	?
A-S		?		?
H-A	Context First	?	Autopsy Second	?
S-A		?		?

Participants were randomly assigned to one of four groups. First, by what contextual information they received, i.e., whether participants received suicide or homicide contextual information. Second, by what order information was presented, i.e., whether they received the contextual or autopsy information first. All participants in all groups received all the four cases. Thus, one group of participants, the A-S group, received the autopsy information first, followed by suicide contextual information. Another group of participants, the S-A group, received the same information but in reverse order, i.e., suicide contextual information first, followed by the autopsy information. The other two groups were the same except that they received the homicide contextual information rather than the suicide: The A-H group, autopsy information first, followed by homicide context; and the H-A group, in reverse order, homicide context first, then the autopsy information (See Table 2).

## 4 Participants

The study used a convenience sample taken from a population of members from a large U.S. based professional forensic pathology organization: the National Association of Medical Examiners (NAME). The solicitation to prospective participants did not reference a professional organization or membership in any professional organization (e.g., NAME, Academy of Forensic Sciences, or International Association of Coroners and Medical Examiners). Moreover, these organizations neither conducted nor endorsed this research.

Potential participants received an email detailing the purpose, objectives, and risks and benefits of the study. Email reminders were sent at the second week and after the fifth week to encourage recruitment and response. No personal identification information was collected, and survey responses were anonymous. Demographic information was collected (see Table 3). The study was approved by the Committee for the Protection of Human Subjects Institutional Review Boards (IRB) at Texas A&M University and deemed exempt.

**Table 3:** Participants demographic information.

	Total n (%)	Group A-S n (%)	Group S-A n (%)	Group A-H n (%)	Group H-A n (%)
**Total**	252	64	72	54	62
**Title**					
Coroner	3 (1.2)	0 (0)	2 (2.8)	1 (1.9)	0 (0)
Medical examiner	35 (13.9)	6 (9.4)	12 (16.7)	5 (9.3)	12 (19.4)
Forensic pathologist	39 (15.5)	8 (12.5)	9 (12.5)	14 (25.9)	8 (12.9)
Death investigator	9 (3.6)	4 (6.3)	1 (1.4)	2 (3.7)	2 (3.2)
Other^a^	11 (4.4)	4 (6.3)	1 (1.4)	3 (5.6)	3 (4.8)
Medical examiner & Forensic pathologist	44 (17.5)	15 (23.4)	10 (13.9)	8 (14.8)	11 (17.7)
Multiple title^b^	10 (4.0)	4 (6.3)	2 (2.8)	1 (1.9)	3 (4.8)
No answer	101 (40.1)	23 (35.9)	35 (48.6)	20 (37.0)	23 (37.1)
**Sex**					
Male	79 (31.3)	25 (39.1)	18 (25.0)	17 (31.5)	19 (30.6)
Female	64 (25.4)	14 (21.9)	18 (25.0)	16 (29.6)	16 (25.8)
Prefer not to answer	7 (2.8)	2 (3.1)	1 (1.4)	1 (1.9)	3 (4.8)
No answer	102 (40.5)	23 (35.9)	35 (48.6)	20 (37.0)	24 (38.7)
**Education**					
High school	0 (0)	0 (0)	0 (0)	0 (0)	0 (0)
Some college associates	2 (0.8)	2 (3.1)	0 (0)	0 (0)	0 (0)
Bachelors	10 (4.0)	6 (9.4)	1 (1.4)	3 (5.6)	0 (0)
Masters	7 (2.8)	1 (1.6)	2 (2.8)	0 (0)	4 (6.5)
PhD	3 (1.2)	2 (3.1)	1 (1.4)	0 (0)	0 (0)
MD	103 (40.9)	24 (37.5)	30 (41.7)	23 (42.6)	26 (41.9)
Prefer not to answer	0 (0)	0 (0)	0 (0)	0 (0)	0 (0)
Other^c^	8 (3.2)	4 (6.3)	1 (1.4)	0 (0)	3 (4.8)
Combined^d^	22 (8.7)	2 (3.1)	3 (4.2)	9 (16.7)	8 (12.9)
No answer	97 (38.5)	23 (35.9)	34 (47.2)	19 (35.2)	21 (33.9)
**What is the total number of years of experience in medicolegal death investigation?**					
1–10 years	46 (18.3)	9 (14.1)	14 (19.4)	10 (18.5)	13 (21.0)
11–20 years	41 (16.3)	8 (12.5)	7 (9.7)	13 (24.1)	13 (21.0)
21–30 years	35 (13.9)	14 (21.9)	8 (11.1)	7 (13.0)	6 (9.7)
31 years or more	24 (9.5)	8 (12.5)	7 (9.7)	4 (7.4)	5 (8.1)
No answer	106 (42.1)	25 (39.1)	36 (50.0)	20 (37.0)	25 (40.3)
**What is the total number of years of experience in your current position?**					
0–10 years	93 (36.9)	20 (31.3)	23 (31.9)	24 (44.4)	26 (41.9)
11–20 years	33 (13.1)	8 (12.5)	8 (11.1)	8 (14.8)	9 (14.5)
21–30 years	11 (4.4)	7 (10.9)	2 (2.8)	0 (0)	2 (3.2)
31 years or more	6 (2.4)	2 (3.1)	3 (4.2)	1 (1.9)	0 (0)
No answer	109 (43.3)	27 (42.2)	36 (50.0)	21 (38.9)	25 (40.3)
**How many examinations involving firearm(s) having you conducted within the last month?**					
0-5	74 (29.4)	18 (28.1)	18 (25.0)	17 (31.5)	21 (33.9)
6-14	50 (19.8)	12 (18.8)	14 (19.4)	12 (22.2)	12 (19.4)
>14	18 (7.1)	6 (9.4)	4 (5.6)	5 (9.3)	3 (4.8)
Other	8 (3.2)	5 (7.8)	1 (1.4)	0 (0)	2 (3.2)
No answer	102 (40.5)	23 (35.9)	35 (48.6)	20 (37.0)	24 (38.7)
**Did law enforcement officers** **Attend and view autopsies of all firearm deaths?**					
Yes	31 (12.3)	11 (17.2)	9 (12.5)	4 (7.4)	7 (11.3)
No	11 (4.4)	2 (3.1)	2 (2.8)	5 (9.3)	2 (3.2)
No, only firearm deaths where the manner is suspected to be homicide	25 (9.9)	6 (9.4)	6 (8.3)	8 (14.8)	5 (8.1)
No, only firearm deaths where the manner is suspected to be suicide	0 (0)	0 (0)	0 (0)	0 (0)	0 (0)
No, only in firearm deaths where the manner is not clear	2 (0.8)	0 (0)	1 (1.4)	0 (0)	1 (1.6)
No, only attend selected firearm -related autopsies depending on circumstances	51 (20.2)	12 (18.8)	13 (18.1)	10 (18.5)	16 (25.8)
Not applicable	1 (0.4)	0 (0)	0 (0)	1 (1.9)	0 (0)
Multiple^e^	22 (8.7)	5 (7.8)	6 (8.3)	5 (9.3)	6 (9.7)
Other^f^	11 (4.4)	5 (7.8)	0 (0)	2 (3.7)	4 (6.5)
No answer	98 (38.9)	23 (35.9)	35 (48.6)	19 (35.2)	21 (33.9)
**Are you responsible for assigning manner of death on the death certificates?**					
Yes	104 (41.3)	21 (32.8)	30 (41.7)	27 (50.0)	26 (41.9)
No	36 (14.3)	15 (23.4)	6 (8.3)	5 (9.3)	10 (16.1)
Not applicable	4 (1.6)	2 (3.1)	0 (0)	1 (1.9)	1 (1.6)
Other	7 (2.8)	3 (4.7)	1 (1.4)	1 (1.9)	2 (3.2)
No answer	101 (40.1)	23 (35.9)	35 (48.6)	20 (37.0)	23 (37.1)
**Who carries out the role of death investigator in your agency?**					
Law enforcement	13 (5.2)	3 (4.7)	7 (9.7)	0 (0)	3 (4.8)
ABMDI qualified death investigator	83 (32.9)	20 (31.3)	17 (23.6)	20 (37.0)	26 (41.9)
Multiple^g^	30 (11.9)	9 (14.1)	7 (9.7)	9 (16.7)	5 (8.1)
Coroner	8 (3.2)	3 (4.7)	1 (1.4)	2 (3.7)	2 (3.2)
Other^h^	17 (6.7)	6 (9.4)	5 (6.9)	3 (5.6)	3 (4.8)
No answer	101 (40.1)	23 (35.9)	35 (48.6)	20 (37.0)	23 (37.1)

a Other includes forensic anthropologist, forensic veterinarian, administrator/forensic anthropologist, retired coroner, retired forensic pathologist, retired medical examiner, forensic pathologist in training.

b Multiple title includes coroner & forensic pathologist, coroner & medical examiner, coroner & forensic pathologist & death investigator, coroner & medical examiner & forensic pathologist, coroner & death investigator, death investigator & director of operations, death investigator & autopsy assistant, and law enforcement & other title.

c Other includes DO, FRCPA.

d Combined education level includes Master's & MD, Master's & PhD, High school & Bachelor's, High school & Bachelor's & MD, Some college associates & Master's, Bachelor's & Master's, Master's & PhD & MD, High School & DO, High School & Doctor of Veterinary Medicine, PhD & MD.

e Multiple includes law enforcement officers will attend autopsies if manner is suspected to be homicide & not clear or depends on circumstances or other manner of death.

f Other includes law enforcement agency, projectile retrieval in police use of force investigations where an officer has shot a dog.

g Multiple includes law enforcement & ABMDI, or law enforcement & coroner, or ABMDI & coroner, or ABMDI qualified death investigator & not ABMDI qualified death investigator.

h Other includes consultant, forensic pathologist, medical examiners, death investigator, in house investigator, non-pathologist physician, M.

A total of 1111 emails were sent to participants. Overall, a total of n = 252 survey recipients responded, for an overall response rate of 22.7% (their distribution among the four groups as follows: Group A-S, n = 64; Group S-A, n = 72; Group A-H, n = 54; and Group H-A, n = 62) (See Table 3). Participants identified themselves as only medical examiners, n = 35 (13.9%); as only forensic pathologists, n = 39 (15.5%); as both medical examiners and forensic pathologists, n = 44 (17.5%); as coroners, n = 3 (1.2%); as having multiple titles, n = 10 (4.0%); as death investigators, n = 9 (3.6%); and as others (which included forensic anthropologists, forensic veterinarian, retired, or forensic pathologist in training, etc.), n = 11 (4.4%). Eighty-three participants (32.9%) were ABMDI qualified. Participants with a MD degree, n = 103 (40.9%), and participants with more than 10 years of experience in medicolegal death investigation, n = 100 (39.7%). In terms of autopsy experience, n = 124 (49.2%) conducted 0–14 examinations specifically involving firearm deaths within the last month before taking part in the study, and n = 31 (12.3%) reported that law enforcement officers attended and viewed autopsies of all firearm deaths.

## 5 Results

Although identical autopsy information was given to all participants across each group, the differing contextual information impacted and shifted the determination of manner of death (see Table 4). For example, in case 1, the *same set* of autopsy information was paired with a suicide context in Groups S-A and A-S, and with a homicide context for groups H-A and A-H. In the suicide context groups, once both autopsy and suicide context information were presented (regardless of order) a total of 41 (28 + 13) determined the case was a suicide, while only 14 (3 + 11) determined it was a homicide. In contrast, in the homicide context groups, when the *same* autopsy information was paired with a context to suggest homicide, a total of 47 (26 + 21) determined the case as a homicide, while only 3 (2 + 1) determined the manner of death as suicide. Other participants reached other conclusions (e.g., 'undetermined') or did not provide a response.

**Table 4:** Participant decisions about manner of death.

	Group A-S n (%)			Group S-A n (%)			Group A-H n (%)			Group H-A n (%)		
	Phase 1 Autopsy		Phase 2 Suicide Context	Phase 1 Suicide Context		Phase 2 Autopsy	Phase 1 Autopsy		Phase 2 Homicide Context	Phase 1 Homicide Context		Phase 2 Autopsy
	64			72			54			62		
**Case 1**												
Suicide	2 (3.1)	28 (43.8)		17 (23.6)	13 (18.1)		1 (1.9)	2 (3.7)		0 (0)	1 (1.6)	
Homicide	22 (34.4)	3 (4.7)		6 (8.3)	11 (15.3)		24 (44.4)	26 (48.1)		30 (48.4)	21 (33.9)	
Accident	3 (4.7)	1 (1.6)		2 (2.8)	1 (1.4)		0 (0)	0 (0)		0 (0)	0 (0)	
Undetermined	12 (18.8)	6 (9.4)		11 (15.3)	10 (13.9)		10 (18.5)	5 (9.3)		9 (14.5)	17 (27.4)	
Natural	0 (0)	0 (0)		1 (1.4)	0 (0)		0 (0)	0 (0)		0 (0)	0 (0)	
Missing	25 (39.1)	26 (40.6)		35 (48.6)	37 (51.4)		19 (35.2)	21 (38.9)		23 (37.1)	23 (37.1)	
**Case 2**												
Suicide	11 (17.2)	33 (51.6)		29 (40.3)	25 (34.7)		12 (22.2)	3 (5.6)		0 (0)	3 (4.8)	
Homicide	10 (15.6)	0 (0)		0 (0)	2 (2.8)		10 (18.5)	25 (46.3)		28 (45.2)	23 (37.1)	
Accident	1 (1.6)	2 (3.1)		1 (1.4)	1 (1.4)		0 (0)	0 (0)		1 (1.6)	0 (0)	
Undetermined	14 (21.9)	0 (0)		4 (5.6)	5 (6.8)		9 (16.7)	3 (5.6)		9 (14.5)	10 (16.1)	
Natural	0 (0)	0 (0)		0 (0)	0 (0)		0 (0)	0 (0)		0 (0)	0 (0)	
Missing	28 (43.8)	29 (45.3)		38 (52.8)	39 (54.2)		23 (42.6)	23 (42.6)		24 (38.7)	26 (41.9)	
**Case 3**												
Suicide	5 (7.8)	14 (21.9)		4 (5.6)	6 (8.3)		3 (5.6)	1 (1.9)		1 (1.6)	0 (0)	
Homicide	14 (21.9)	0 (0)		0 (0)	3 (4.2)		16 (29.6)	18 (33.3)		16 (25.8)	11 (17.7)	
Accident	3 (4.7)	11 (17.2)		9 (12.5)	6 (8.3)		0 (0)	8 (14.8)		11 (17.7)	9 (14.5)	
Undetermined	13 (20.3)	9 (14.1)		18 (25.0)	17 (23.6)		11 (20.4)	3 (5.6)		8 (12.9)	13 (21.0)	
Natural	0 (0)	0 (0)		1 (1.4)	0 (0)		0 (0)	0 (0)		0 (0)	0 (0)	
Missing	29 (45.3)	30 (46.9)		40 (55.6)	40 (55.6)		24 (44.4)	24 (44.4)		26 (41.9)	29 (46.8)	
**Case 4**												
Suicide	0 (0)	19 (29.7)		19 (26.4)	15 (20.8)		0 (0)	0 (0)		0 (0)	0 (0)	
Homicide	20 (31.3)	2 (3.1)		1 (1.4)	4 (5.6)		26 (48.1)	30 (55.6)		21 (33.9)	27 (43.5)	
Accident	4 (6.3)	2 (3.1)		1 (1.4)	2 (2.8)		0 (0)	0 (0)		0 (0)	0 (0)	
Undetermined	9 (14.1)	8 (12.5)		11 (15.3)	11 (15.3)		4 (7.4)	0 (0)		11 (17.7)	5 (8.1)	
Natural	0 (0)	0 (0)		0 (0)	0 (0)		0 (0)	0 (0)		0 (0)	0 (0)	
Missing	31 (48.4)	33 (51.6)		40 (55.6)	40 (55.6)		24 (44.4)	24 (44.4)		30 (48.4)	30 (48.4)	

The same reliance on context, relative to the autopsy findings, was seen for each case scenario. For case 2, the suicide context in Groups A-S and S-A led 58 (33 + 25) participants to determine the manner of death as suicide in phase 2, with only 2 (0 + 2) participants determining it as homicide. Conversely, when the same set of autopsy information was paired with a homicide context in Groups A-H and H-A, only 6 (3 + 3) participants determined the manner to be suicide, while 48 (25 + 23) participants determined it to be homicide. For case 3, autopsy information with suicide context in Groups A-S and S-A led 20 (14 + 6) participants to determine the manner was suicide, and only 3 (0 + 3) as homicide; the same set of autopsy information paired with a homicide context in Groups A-H and H-A resulted in 29 (18 + 11) respondents determining the case as homicide and only 1 (1 + 0) participant determining it was a suicide. Finally, comparable results were also seen in case 4: when the autopsy information was paired with a suicide context in Groups A-S and S-A, 34 (19 + 15) participants determined the manner of death was suicide, and only 6 (2 + 4) as homicide. Conversely, when the same set of autopsy information was paired with a homicidal context in Groups A-H and H-A, no participant concluded the manner of death was suicide, while a striking 57 (30 + 27) participants determined the case as homicide (Table 4).

The *final* manner of death decisions further shows the impact of the contextual information across all four cases, X^2^ = 243.43, p < .001. In the suicide groups (A-S and S-A), 153 participants determined the manner of death as suicide across the four cases; in contrast, in the homicide groups (A-H and H-A), only 10 participants determined the manner of death as suicide. Conversely, in the homicide groups participants rendered 181 determinations of homicide across the four cases, in contrast to the suicide groups, in which only 25 participants determined the cases as homicides –see Table 5.

**Table 5:** Participants final manner of death decisions (suicide vs. homicide only) across the four cases, by the contextual information they received (suicide vs. homicide), X^2^ = 243.43, p < .001).

		**Contextual Information**	
		Suicide	Homicide
**Final Decision**	Suicide	153	10
	Homicide	25	181

To examine the level of impact of different information as a function of presentation order, the change in the manner of death decisions (between phase 1 and phase 2 –see Table 2) was calculated for each group. This measurement further reflected the influence and impact of each type of information: If the more impactful information is presented first, then additional information will only minimally change the initial decision based on the more impactful information. However, if the more impactful information is presented later, then its strong impact will override the previous, less impactful information, and the initial decision will therefore be more readily changed.

The data show that when the context is presented first, the rate of change is 16.3%. However, the rate of change increases to 45.4%, when the context is presented second (see Table 6). For example, in death case 1 in Group A-S, when the autopsy information was first presented prior to the suicide contextual information, only 2 participants determined the case was a suicide, and 22 as a homicide. However, once the suicide contextual information was presented, 28 (of those same participants) now determined the manner of death was suicide, and only 3 now determined the case was a homicide. Conversely, when the context was presented prior to autopsy findings, in Group S-A, 17 participants determined the case as a suicide, while only 6 determined it was homicide; once the autopsy information was presented, 13 of the same respondents decided the manner was suicide, while 11 considered the case a homicide.

**Table 6:** Changes in decisions as a function of when participants received contextual information. Participants in groups H-A and S-A received the context before the autopsy information, and participants in groups A-H and A-S received the context after the autopsy information. The latter changed their decisions much more than the former.

			Death Cases				Mean
			1	2	3	4	
GROUPS	Context ***First***	H-A	23.1% (18/78)	13.5% (10/74)	18.8% (13/69)	18.8% (12/64)	18.6%
		S-A	16.7% (12/72)	10.4% (7/67)	15.6% (10/64)	12.5% (8/64)	13.9%
		Mean Context *First*	20.0%	12.1%	17.3%	15.6%	**16.3%**
	Context ***Second***	A-H	8.8% (8/68)	48.4% (30/62)	33.3% (20/60)	13.3% (8/60)	26.4%
		A-S	68.8% (53/77)	66.2% (47/71)	50.7% (35/69)	62.5% (40/64)	62.3%
		Mean Context *Second*	42.1%	57.9%	42.6%	38.7%	**45.4%**

The total percentage change in decision for each group and case considered all five manners and the summary of the findings is captured in Table 6. For example, in case 1, Group A-S, there are total of 77 responses, and changes in decisions occurred 26 times for suicide, 19 times for homicide, 2 times for accidental and 6 times for undetermined. The total percentage of change in decision for death case 1, Group A-S, is 68.8% (53/77). Overall our results can be characterized by a higher rate of change in decisions about manner of death when context was presented after the autopsy information.

## 6 Discussion

Manner of death is a determination that is often based on synthesis of information. Our data demonstrated the power of contextual information, showing that its impact –at least in these cases, concerning single gunshot wounds– was so significant that participants tended to rely on context more than on autopsy information in determining manner of death. This held true regardless of the order of presentation, as participants changed initial autopsy-based conclusions to align with the contextual information. This raises important questions about what, how, and when information should be considered in manner of death determinations.

The impact of contextual information and the order of information presentation may well depend on the level at which the autopsy information contributes to the manner of death determination. When, for example, autopsy information aids relatively little in determining manner of death (as in these cases with single gunshot wounds), then the non-autopsy contextual information has a predominate impact: so much so, that the order in which information is presented has little-to-no impact. In such cases, the contextual information is so powerful that it overrides the autopsy information regardless of order. Similarly, when the autopsy information clearly reflects the manner of death, the role and impact of the order of presenting contextual information is also minimized. However, when interpreting the autopsy findings requires a moderate level of contextual input, or when different sources of information suggest different manners of death, then the order of presentation is more important.

Contextual information plays such an important role in manner of death determination that those responsible for determining manner of death can (and should) explore methods to assure that context is gathered, presented, and considered in ways that will enhance their decisions. Order of information does not only determine what is best remembered, but also creates different expectations and what hypotheses are generated, all of which impact how subsequent information is perceived and interpreted.

Since the order of information is important, and since forensic pathologists need to examine information in some order, Linear Sequential Unmasking - Expanded (LSU-E) suggests using criteria to prioritize and optimize the order: Objectivity, relevance and biasability [11,12]. The idea is not to deprive forensic practitioners from information they need, but to reflect on the order they consider information.

Transparency is also critical and prescribed by methods such as LSU-E: not only specifying and evaluating what information was considered, and how it contributed to a manner of death determination, but also (given people's limited ability to introspect and correctly account for their thinking processes, a meta-cognitive issue) documenting when and what information was available to the decision-maker. Although the autopsy report is a convenient place to include such information, the death certificate affords minimal opportunity to expand upon its classifications; rather, manner of death is simply a checkbox with no attached explanation, increasing the potential for misunderstandings.

It is important to note that although our sample size was considerable, the participants were heterogeneous in their expertise (background, training, and experiences). Nevertheless, expertise does not give protection from the influence of contextual influence or confirmation bias [8,13]. Although we focused primarily on the participants' homicide vs. suicide decisions, it is important to note that many participants reached a decision of 'undetermined' or did not provide a response. In addition, our study –focused specifically on manner of death determination– does not include the full scope of the tasks incumbent upon the forensic practitioner during casework (e.g., evidence collection, prioritization, organ/tissue donation requests, etc.) which should also be considered in any discussion of information sequencing.

The influence of contextual information on manner of death determination must be recognized; further research is needed to explore the interplay between different sources of information and their impact on manner of death decisions, as well as how the degree of trustworthiness of different sources of information may influence decisions. We strongly encourage collaborations such as this, between forensic practitioners and cognitive researchers, which are required to understand the nature of decision-making in medicolegal death investigation and to develop ways to improve and enhance forensic decisions.

## Declaration of competing interest

No conflict of interest.
